# Number of embryos transferred and diagnosis of preeclampsia

**DOI:** 10.1186/s12958-020-00627-7

**Published:** 2020-07-11

**Authors:** Cynthia K. Sites, Donna Wilson, Dana Bernson, Sheree Boulet, Yujia Zhang

**Affiliations:** 1 Department of Obstetrics and Gynecology, University of Massachusetts Medical School, Baystate, 759 Chestnut Street, S1683, Springfield, MA 01199 USA; 2 Department of Epidemiology and Biostatistics, Baystate Medical Center, Springfield, MA USA; 3 Department of Public Health, Boston, MA USA; 4 Department of Gynecology and Obstetrics, Emory University School of Medicine, Atlanta, GA USA; 5 Centers for Disease Control and Prevention, Atlanta, GA USA

**Keywords:** Assisted reproductive technology, Cryopreserved embryos, Preeclampsia, Singleton gestation, Twin gestation, Vanishing twin

## Abstract

**Background:**

Multiple births and first pregnancy are associated with higher preeclampsia risk. It is unknown if the transfer of multiple embryos or first embryo transfer with assisted reproductive technology (ART) is also associated with greater preeclampsia risk.

**Methods:**

We performed a retrospective cohort study of IVF clinics and hospitals in Massachusetts. We used linked ART surveillance, birth certificate, and maternal hospitalization discharge data for 21,188 births, considering resident singleton (12,810) and twin (8378) live-births from autologous or donor eggs from 2005 to 2012. We used log binomial and Poisson regression to calculate adjusted relative risks (aRRs) and 95% confidence intervals (CI) for the association between preeclampsia and predictors of preeclampsia. Outcomes were stratified by singleton and twin birth, donor versus autologous cycles, and use of fresh versus cryopreserved embryos.

**Results:**

Considering all singleton births, the transfer of multiple embryos increased the risk of preeclampsia [aRR = 1.10 (95% CI: 1.01–1.19)]. Relative risks were greatest for fresh non-donor cycles [aRR = 1.14 (95% CI: 1.03–1.26)]. Vanishing twin and number of prior ART cycles was not associated with preeclampsia among singleton births [aRR = 1.18 (95% CI: 0.91–1.53)], and aRR = 1.01 (95% CI: 0.96–1.05)], respectively. Considering all twin births, the transfer of > 2 embryos increased the risk of preeclampsia [aRR = 1.09 (95% CI: 1.001–1.19)]. Vanishing triplet and number of prior ART cycles were not associated with preeclampsia among twin births [aRR = 0.93 (95% CI: 0.69–1264), and aRR = 0.98 (CI: 0.95–1.02)], respectively.

**Conclusions:**

Among ART births, the transfer of more than 1 embryo for singleton gestations and more than 2 embryos for twin gestations increased the risk for preeclampsia diagnosis.

## Background

Preeclampsia is defined by the American College of Obstetrics and Gynecology as maternal hypertension with end organ injury after 20 weeks’ gestation [1]. The incidence of preeclampsia is increasing worldwide, with manifestations that include proteinuria, thrombocytopenia, renal insufficiency, impaired liver function, pulmonary edema, visual symptoms, and maternal and fetal mortality [1].

The use of Assisted Reproductive Technology (ART) is reported to increase preeclampsia risk by 16% compared with spontaneous conception [2]. Other risk factors for preeclampsia include advanced maternal age, nulliparity, non-Hispanic Black race/ethnicity, obesity, multiple gestation, egg donor pregnancy, male infant, prior preeclampsia, chronic hypertension, diabetes mellitus, and embryo cryopreservation [1, 3, 4].

The increased rate of preeclampsia among twin births compared with singletons may be due to a larger placental mass or relative placental ischemia in twin gestations compared with singletons [5]. The transfer of multiple embryos often results in twins, but it is unknown if the transfer of multiple embryos increases the risk for preeclampsia.

Vanishing twin, or fetal resorption, is a fetus in a multi-gestation pregnancy that dies in utero, usually early in pregnancy, and is partially or completely reabsorbed [6]. The surviving infant in a vanishing twin ART pregnancy may be small for gestational age [7], or have greater risk for preterm delivery [8, 9] compared to ART pregnancies that were originally singletons. To date, we are unaware of prior reports of the effect of a vanishing twin on preeclampsia risk.

With spontaneous conceptions, the initial pregnancy is at greater risk for preeclampsia compared to subsequent pregnancies, 4.1% vs. 1.7%, which could be related to reduced prior exposure to paternal or fetal antigens in first pregnancies compared to those that follow [10, 11]. We are aware of only one small study considering the risk for preeclampsia in the initial ART cycle versus subsequent ART cycles, showing no difference between groups [12].

We hypothesized that preeclampsia diagnosis would be increased in singleton and twin gestations if more embryos were transferred compared with one embryo for singletons or two embryos for twins, and that the finding of vanishing twin would increase preeclampsia risk. Furthermore, we hypothesized that preeclampsia would be increased in the first ART cycle compared with subsequent ART cycles.

## Methods

We used linked data from the States Monitoring ART (SMART) Collaborative, a project whereby the Centers for Disease Control and Prevention (CDC) links data from the National Assisted Reproductive Technology Surveillance System (NASS) to maternal hospital discharge and vital records from State Departments of Health in Connecticut, Massachusetts, and Michigan. Details about this linked data have been described previously [13, 14]. During the time of this analysis, only Massachusetts had all variables necessary for the analysis in hospital discharge data, so data from Connecticut and Michigan were excluded. The linkage rate was 88.5% for Massachusetts from 2005 to 2012.

We included resident births from ART in Massachusetts from 2005 to 2012 at > 20 weeks gestational age. Our study was approved by the Institutional Review Boards of the CDC and the Massachusetts Department of Public Health, and was deemed exempt by the Institutional Review Board at Baystate Medical Center.

We compared demographic and clinical characteristics of singleton and twin births, stratified by cycle type (autologous vs. donor egg, and fresh vs. cryopreserved embryo). Demographic and clinical variables were obtained from the NASS database (gravidity, parity, body mass index [BMI] at the start of the ART cycle, embryo transfer type, number of pregnancy sacs seen by initial ultrasound), birth certificates (number of infants born, gestational age, infant sex, mother’s age and race/ethnicity), and maternal hospital discharge information. Cells with *n* < 5 in tables were suppressed to protect confidentiality, per CDC and MA State Department of Health requirements.

We used ICD-9-CM codes from maternal hospital discharge information to identify the diagnosis of preeclampsia without and with severe features (642.40–642.54, and 642.50–642.5), pregestational diabetes (648.00), gestational diabetes (648.8), chronic hypertension (401.90) and gestational hypertension (642.0–642.04). An additional variable to identify women diagnosed with both preeclampsia and preterm (< 37 weeks) delivery was created by combining preeclampsia and preterm delivery information, as described previously [4]. We could not determine when the diagnosis of preeclampsia was made during gestation in this database.

Vanishing twin or vanishing triplet was identified if the number of fetal heartbeats by ultrasound at 6–8 weeks estimated gestational age was greater than the number of infants born [6]. If early ultrasound information was missing, cases were excluded (0.5% of singletons and 0.4% of twin births). The number of records dropped from the models due to missing data was low (< 1% for singleton births and < 2% for twin births). Maternal demographic variables were missing in < 1% of records, with the exception of maternal BMI, which was missing in 47% of cases. Mothers of twins were included only once in the analysis.

Chi-square and Fisher’s exact tests were used for bivariate comparisons. Continuous variables were compared with unpaired t-tests. We used log binomial and Poisson regression to calculate associations between preeclampsia and possible predictors (number of embryos transferred, finding of a vanishing twin or triplet, or prior ART cycles), stratified by singleton and twin birth and accounting for correlated responses of women with more than one record. Approximately 3% of singleton births had more than one record [15].

Results were calculated for all births and stratified by use of autologous vs. donor eggs and fresh vs. cryopreserved embryos due to heterogeneity of relationships between preeclampsia and predictors by cycle type. Covariates in the models were gravidity, infant sex, mother’s age, non-white race, maternal diabetes, and maternal hypertension. Two-tailed probabilities of < 0.05 were considered as significant. All analyses were conducted using SAS (SAS Institute, Cary, NC, USA).

## Results

Maternal demographic and clinical characteristics are presented in Table 1. Our sample of 21,188 births from ART (12,810 singleton and 8378 twin) included a majority of patients who were nulliparous and Non-Hispanic White. Women having donor egg cycles were older than those having non-donor egg cycles.

**Table 1:** Demographic and clinical characteristics of singleton and twin births by oocyte source and embryo type

Characteristic N (%)	Singleton Birth				Twin Birth			
	Autologous		Donor		Autologous		Donor	
	Fresh (*N* = 10,137)	Cryopreserved (*N* = 1557)	Fresh (*N* = 861)	Cryopreserved (*N* = 363)	Fresh (*N* = 6742)	Cryopreserved (*N* = 809)	Fresh (*N* = 855)	Cryopreserved (*N* = 142)
Age, years (mean ± S.D.)	35.3 (4.1)	35.1 (3.9)	41.9 (4.4)	42.4 (4.4)	34.6 (3.8)	34.4 (3.8)	41.3 (4.8)	43.1 (4.1)
Infant sex: Male	5257 (51.9)	802 (51.5)	438 (50.9)	180 (49.6)	3435 (51.0)	429 (53.0)	433 (50.6)	75 (52.8)
Gravida = 1	4638 (45.8)	488 (31.4)	377 (44.1)	81 (22.5)	3263 (48.4)	305 (38.0)	373 (43.8)	38 (26.8)
Parity = 0	6834 (67.8)	784 (50.8)	627 (73.7)	187 (53.1)	4657 (69.5)	477 (59.9)	603 (71.4)	74 (52.1)
Body Mass Index (kg/m^2^)								
< 18.5	129 (2.4)	25 (3.1)	8 (1.9)	< 5	98 (2.7)	6 (1.4)	10 (2.7)	< 5
18.5–24.9	3245 (59.1)	454 (56.2)	273 (63.3)	92 (55.8)	2092 (58.9)	256 (58.5)	246 (66.5)	38 (63.3)
25–25.9	1260 (22.9)	203 (25.1)	96 (22.3)	44 (44 (26.7)	798 (22.5)	106 (24.2)	88 (23.8)	12 (20.0)
> 30	859 (15.6)	126 (15.6)	54 (12.5)	27 (16.4)	567 (16.0)	70 (16.0)	26 (7.0)	8 (13.3)
Missing (N)	4644	749	430	198	3187	371	495	82
Race/Ethnicity								
White, non-Hispanic	8428 (83.3)	1280 (82.2)	732 (85.3)	318 (87.6)	5641 (83.9)	671 (83.6)	765 (89.9)	114 (80.3)
Black, non-Hispanic	327 (3.2)	65 (4.2)	26 (3.0)	10 (2.8)	182 (2.7)	36 (4.5)	20 (2.4)	6 (4.2)
Hispanic	406 (4.0)	66 (4.2)	36 (4.2)	14 (3.9)	292 (4.3)	46 (5.7)	26 (3.1)	< 5
American Indian	90 (0.9)	12 (0.8)	< 5	< 5	68 (1.0)	7 (0.9)	6 (0.7)	< 5
Asian/Pacific Islander	869 (8.6)	134 (8.6)	57 (6.6)	20 (5.5)	542 (8.1)	43 (5.4)	34 (4.0)	20 (14.1)
Chronic hypertension	113 (1.1)	25 (1.6)	23 (2.7)	7 (1.9)	102 (1.5)	6 (0.7)	30 (3.5)	6 (4.2)
Gestational Hypertension	476 (4.7)	93 (6.0)	63 (7.3)	39 (10.7)	465 (6.9)	86 (10.6)	88 (10.3)	8 (5.6)
Diabetes	92 (0.9)	16 (1.0)	5 (0.6)	< 5	48 (0.7)	< 5	< 5	< 5
Gestational Diabetes	746 (7.4)	123 (7.9)	80 (9.3)	31 (8.5)	665 (9.9)	60 (7.4)	98 (11.5)	26 (18.3)
Infertility Diagnosis								
Tubal	819 (8.1)	135 (8.7)	8 (0.9)	6 (1.7)	472 (7.0)	85 (10.5)	6 (0.7)	< 5
Ovulatory dysfunction	849 (6.6)	155 (10.0)	17 (2.0)	20 (5.5)	619 (9.2)	95 (11.7)	34 (4.0)	10 (7.0)
Diminished ovarian reserve	455 (4.5)	25 (1.6)	404 (46.9)	140 (38.6)	220 (3.3)	6 (0.7)	372 (43.5)	52 (36.6)
Endometriosis	415 (4.1)	61 (3.9)	11 (1.3)	< 5	273 (4.1)	34 (4.2)	14 (1.6)	6 (4.2)
Uterine	116 (1.1)	19 (1.2)	< 5	< 5	70 (1.0)	6 (0.7)	< 5	< 5
Male only	2441 (24.1)	382 (24.5)	18 (2.1)	< 5	1610 (23.9)	198 (24.5)	36 (4.2)	< 5
Other	879 (8.7)	116 (7.5)	137 (15.9)	81 (22.3)	572 (8.5)	74 (9.2)	163 (19.1)	34 (23.9)
Unknown	2532 (25.0)	388 (24.9)	54 (6.3)	25 (6.9)	1847 (27.4)	205 (25.3)	66 (7.7)	10 (7.0)
Multiple female w/o male	512 (5.1)	98 (6.3)	88 (10.2)	40 (11.0)	317 (4.7)	24 (3.0)	76 (8.9)	14 (10.0)
Multiple female + male	1119 (11.0)	178 (11.4)	122 (14.2)	40 (11.0)	742 (11.0)	82 (10.1)	86 (10.1)	8 (5.6)

Cells with *N* < 5 were suppressed to protect confidentiality

Table 2 depicts the frequencies of preeclampsia and of preeclampsia occurring with preterm delivery in each group, along with preeclampsia predictors (number of embryos transferred, number of prior ART cycles, and vanishing twin or triplet). In non-donor cycles, women having cryopreserved transfers were more likely to be diagnosed with preeclampsia or preeclampsia occurring with preterm delivery compared with those having fresh transfers. Overall, the mean number of embryos transferred was similar between groups. Women having prior ART cycles were more likely to have cryopreserved transfers compared to fresh transfers. Vanishing twins or triplets occurred in approximately 7% of cycles.

**Table 2:** Predictors of preeclampsia in singleton and twin births by oocyte source and embryo type

	Singleton Birth				Twin Birth			
	Autologous		Donor		Autologous		Donor	
Variable N (%)	Fresh (*N*=10137)	Cryopreserved (*N*=1557)	Fresh (*N*=861)	Cryopreserved (*N*=363)	Fresh (*N*=6742)	Cryopreserved (*N*=809)	Fresh (*N*=855)	Cryopreserved (*N*=142)
Preeclampsia	468 (4.6)	114 (7.3)	105 (12.2)	32 (8.8)	1148 (17.0)	172 (21.3)	230 (26.9)	42 (29.6)
Preeclampsia with preterm delivery	168 (1.7)	41 (2.6)	37 (4.3)	14 (3.9)	832 (12.3)	129 (16.0)	166 (19.4)	34 (23.9)
# of embryos transferred								
1	2006 (19.8)	453 (29.1)	152 (17.7)	99 (27.3)	70 (1.0)	22 (2.7)	<5	<5
2	5614 (55.4)	802 (51.5)	674 (78.3)	212 (58.4)	4744 (70.4)	559 (69.1)	806 (94.3)	104 (73.4)
3+	2517 (24.8)	302 (19.4)	35 (4.1)	52 (14.3)	1928 (28.6)	228 (28.2)	45 (5.3)	34 (23.9)
# embryos transferred (mean ± S.D.)	2.2 (1.0)	2.0 (0.8)	1.9 (0.5)	1.9 (0.7)	2.4 (0.9)	2.4 (0.8)	2.1 (0.3)	2.3 (0.6)
# prior ART cycles								
0	5240 (51.7)	249 (16.0)	362 (42.0)	43 (11.9)	3464 (51.4)	107 (13.2)	340 (39.8)	12 (8.5)
1+	4896 (48.3)	13.8 (84.0)	499 (58.0)	320 (88.2)	3278 (48.6)	702 (86.8)	515 (60.2)	130 (91.6)
Vanishing twin/triplet	864 (8.5)	88 (5.8)	82 (9.6)	26 (7.4)	395 (5.9)	32 (4.1)	13 (1.5)	10 (7.3)

The results of the adjusted binomial and Poisson regression models for preeclampsia among singletons is shown in Table 3. Considering all singleton births, with the transfer of each additional embryo beyond 1, the risk of preeclampsia increased by 10%. Among births from fresh autologous embryos, number of embryos transferred was associated with preeclampsia [adjusted risk ratio (aRR) = 1.14 (95% confidence interval (CI): 1.03–1.26)]. Among singleton births from fresh donor egg transfer, preeclampsia risks were increased for those who had a prior ART cycle vs. a first ART cycle [aRR = 1.07 (95% CI: 1.001–1.14]. Preeclampsia was not associated with vanishing twin [aRR = 1.18 (95% CI: 0.91–1.53)], or with having a prior ART cycle when all singleton cycle types were considered together [aRR = 1.01 (95% CI: 0.96–1.05)].

**Table 3:** Adjusted^a^ multivariable log binomial and Poisson regression models: single birth preeclampsia relative risk

	All cycles ***N***=12,810		Fresh Autologous ***N***=10,110		Cryopreserved Autologous ***N***=1,502		Fresh Donor ***N***=849		Cryopreserved Donor ***N***=349	
	aRR (CI)	*p* value	aRR (CI)	*p* value	aRR (CI)	*p* value	aRR (CI)	*p* value	aRR (CI)	*p* value
# embryos transferred (continuous)	1.10 (1.01, 1.19)	0.045	1.14 (1.03, 1.26)	0.018	1.14 (0.90, 1.44)	0.309	0.80 (0.56, 1.15)	0.222	0.90 (0.58, 1.39)	0.628
# prior ART cycles (continuous)	1.01 (0.96, 1.05)	0.724	0.99 (0.92, 1.05)	0.659	0.96 (0.86, 1.08)	0.495	1.07 (1.001, 1.14)	0.038	0.98 (0.87, 1.11)	0.742
Vanishing twin (yes vs. no)	1.18 (0.91, 1.53)	0.268	1.03 (0.72, 1.44)	0.911	1.62 (0.84, 3.14)	0.236	1.35 (0.79, 2.33)	0.277	1.31 (0.40, 4.32)	0.697

^a^controlling for cycle type (all cycles model only), gravida, infant sex, mother's age, non-White race, diabetes and hypertension

*Referent values:*

Total embryos: Continuous

Prior ART: Continuous

Vanishing twin: No

Cycle type: Fresh non-donor

Gravida: Continuous

Infant sex: Male

Mother's age: Continuous

Non-White race: White

Diabetes: No

Hypertension: No

The results of the adjusted binomial and Poisson regression model for preeclampsia among twin births is shown in Table 4. Considering all cycles, with each additional embryo transferred greater than 2, the risk of preeclampsia increased by 9%. However, there was no association with the finding of a vanishing triplet [aRR = 0.93 (95% CI: 0.69–1.26)]. History of prior ART cycles had no effect on preeclampsia diagnosis among twin gestations considered together or as separate groups.

**Table 4:** Adjusted^a^ multivariable log binomial and Poisson regression models: twin birth preeclampsia relative risk

	All cycles ***N***=8,378		Fresh Autologous ***N***=6,650		Cryopreserved Autologous ***N***=753		Fresh Donor ***N***=841		Cryopreserved Donor ***N***=134	
	aRR (CI)	*p* value	aRR (CI)	*p* value	aRR (CI)	*p* value	aRR (CI)	*p* value	aRR (CI)	*p* value
# embryos transferred (continuous)	1.09 (1.001, 1.19)	0.040	1.07 (0.98, 1.17)	0.137	1.03 (0.84, 1.28)	0.722	0.58 (0.24, 1.41)	0.232	1.49 (0.69, 3.19)	0.300
# prior ART cycles (continuous)	0.98 (0.95, 1.02)	0.406	0.99 (0.96, 1.02)	0.701	1.13 (0.99, 1.28)	0.062	0.97 (0.89, 1.06)	0.548	1.01 (0.89, 1.16)	0.854
Vanishing triplet (yes vs. no)	0.93 (0.69, 1.26)	0.639	0.93 (0.78, 1.11)	0.453	0.86 (0.32, 2.31)	0.766	1.53 (0.42, 5.52)	0.519	0.28 (0.01, 7.76)	0.451

^a^controlling for cycle type (all cycles model only), gravida, infant sex, mother's age, non-White race, diabetes and hypertension

*Referent values:*

Total embryos: Continuous

Prior ART: Continuous

Vanishing twin: No

Cycle type: Fresh non-donor

Gravida: Continuous

Infant sex: Male

Mother's age: Continuous

Non-White race: White

Diabetes: No

Hypertension: No

## Discussion

We report that the transfer of > 1 embryo with singleton gestations and > 2 embryos with twin gestations increased the risk of preeclampsia diagnosis. The transfer of multiple embryos may result in multiple implantations with an increased placental mass, or relative placental ischemia, which could increase preeclampsia risk [5]. However, we did not find that the presence of a vanishing twin or triplet by early ultrasound increased the risk of preeclampsia, which may be related to the definition of vanishing twin, which requires fetal cardiac activity by ultrasound [6]. We hypothesized that women having their first ART cycle would have greater risk of preeclampsia than those having subsequent cycles due to reduced exposure to new paternal or fetal antigens, but our data did not support this.

It is biologically plausible that early implantations that do not progress to viability with fetal cardiac activity could increase preeclampsia risk. One theory of preeclampsia development involves greater genetic or immune incompatibility between mother and fetuses in dichorionic twin gestations compared to monochorionic twin or singleton gestations [16]. Maternal natural killer cells may interact with paternal human leukocyte antigens, with a cytotoxic T-cell response leading to the development of preeclampsia [17, 18]. In twin gestations, women are exposed to a greater number of fetal antigens compared to women with singleton gestations, with the possibility of triggering a greater immune response, increasing the development of preeclampsia. Our database was unable to identify early gestational sacs (implantations) before viability was confirmed by ultrasound, which may have contributed to increased preeclampsia risk when multiple embryos were transferred.

An effect of vanishing twin on early placentation has been reported previously. Specifically, the surviving infant of a vanishing twin ART pregnancy is reported to be smaller for gestational age [7], and be more likely to deliver preterm [8, 9] compared with ART gestations that were originally singletons. In addition, the later that the vanishing twin occurred in gestation, the greater the risk for the singleton survivor to have a birthweight < 2500 g [7]. Furthermore, preterm and extremely preterm delivery were increased with singleton survivors, especially with late vanishing twin pregnancies [9]. In our study, we compared fetal heartbeats by ultrasound early in gestation to number of infants born, and were unable to determine specifically when the vanishing twin or triplet occurred in gestation after viability was confirmed.

The incidence of vanishing twin/triplet is reported to occur in 50% of pregnancies that have at least three gestational sacs initially, and in 36% of pregnancies with two gestational sacs initially [19]. Among ART conceptions, it may occur in 12–30% of conceptions [20]. Our 7% overall incidence of vanishing twin with ART was somewhat lower than 12–30%, possibly due to the trend toward elective single embryo transfer in recent years in Massachusetts.

We found that cryopreserved autologous embryo transfer with singleton birth resulted in a 1.59-fold increase in preeclampsia compared to fresh transfer with singleton birth. During this time period in Massachusetts, endometrial preparation for cryopreserved embryo transfers was usually “artificial” or with hormone replacement therapy rather than by natural corpus luteum hormone production or by “natural” cycle. A recent study reported an association between the absence of a corpus luteum, as would be seen with artificial or hormone replacement therapy, and higher rates of preeclampsia, compared to natural cycle frozen embryo transfer [21]. It is possible that our finding of a higher incidence of preeclampsia diagnosis with cryopreserved autologous transfers compared to fresh transfers is related to absence of a corpus luteum with these cryopreserved embryo transfers [22]. Our finding of similar rates of preeclampsia in cryopreserved and fresh embryo transfers following donor egg cycles, where no corpora lutea were likely present in either type of cycle, support this hypothesis. Our database did not include information about endometrial preparation, so we cannot confirm this finding.

Our finding that the first embryo transfer did not increase preeclampsia risk compared to subsequent embryo transfers is similar to a smaller study of 364 patients that reported no differences in preeclampsia rates between those who conceived following the first embryo transfer and those who conceived after two or more transfers [12]. Our finding of a small increased risk for preeclampsia in singleton fresh donor egg gestations following subsequent embryo transfer compared to first embryo transfer was unexpected. Although we adjusted for maternal age and other known confounders, there could be residual confounding, or this finding may have been due to chance.

Our study had several strengths as well as a few limitations. We considered a large database, with high linkage of all ART cases to hospital and vital records of infant deliveries in a single state. We controlled for multiple confounders of preeclampsia diagnosis in the multivariable models. Nearly all pregnancies had early ultrasounds to detect fetal heart beats, with < 1% excluded for missing early ultrasounds. However, we were unable to determine in which trimester the vanishing twin/triplet occurred in our study, and were unable to determine if a gestational sac was present without cardiac activity by ultrasound. The definition of prior ART cycles included cycles with a prior embryo transfer, but could not determine whether these prior cycles were fresh or cryopreserved-thawed. In addition, we had no information about history of preeclampsia in prior pregnancies, and we assume that the birth certificate estimate of gestational age accurately reflects the known gestational age based on the date of embryo transfer. Finally, there may be ICD-9-CM coding errors for diagnosis of preeclampsia among different providers. Women with a diagnosis of chronic hypertension (642.7) or gestational hypertension (642.0–642.04) may have been classified as having preeclampsia, but we considered these diagnoses and controlled for them in our analysis as coded.

## Conclusions

The policy of single embryo transfer, in addition to its known reduction in multiple births and prematurity, may also reduce preeclampsia risk. Given the increased trend toward performing cryopreserved embryo transfers, it is relevant that we did not find that transferring more cryopreserved embryos was correlated to preeclampsia diagnosis. Understanding how increasing single embryo transfer, in addition to its known reduction in multiple births and prematurity, may also reduce the risk for preeclampsia, is important for patient and provider awareness and care delivery policies.

## Data Availability

Not applicable.

## Abbreviations

ART: Assisted reproductive technology
aRRs: Adjusted relative risks
CI: Confidence interval
SMART: States Monitoring Assisted Reproductive Technology
CDC: Centers for Disease Control and Prevention
NASS: National Assisted Reproductive Technology Surveillance System
ICD-9-CM: Official system of assigning codes to diagnoses and procedures in the United States
BMI: Body mass index (kg/m2)
SAS: Analytics Software and Solutions
